# Use of community-based surveillance to enhance emerging infectious disease intelligence generation in Indonesia

**DOI:** 10.7189/jogh.15.04118

**Published:** 2025-06-20

**Authors:** Adam T Craig, Andrew Prasettya Japri, Bambang Heryanto

**Affiliations:** 1 The University of Queensland, Centre for Clinical Research, Brisbane, Australia; 2 Australia-Indonesia Health Security Partnership, Jakarta, Indonesia; 3 Independent Public Health Consultant, Jakarta, Indonesia

## Abstract

**Background:**

Community-based surveillance (CBS) refers to a structured process whereby community members systematically detect and report events of public health significance occurring within their communities. In response to the COVID-19 pandemic, the Government of Indonesia has taken steps to enhance national preparedness for future health threats, focussing on expanding the use of CBS. Given the novelty of CBS’s use in Indonesia, we undertook this research to provide evidence-based advice to guide its implementation across the country’s diverse communities and operational contexts.

**Methods:**

We employed three strategies to gather data: a content analysis of policy documents related to CBS implementation in Indonesia, site visits, and interviews with purposefully selected key informants from the animal, wildlife and human health sectors, planning agencies, village leadership, and inter-government coordination bodies. We conducted additional interviews with staff from the United Nations and donor development assistance agencies involved in CBS implementation in Indonesia. A semi-structured tool guided the interviews, and we analysed the data using an inductive approach.

**Results:**

We identified eight policy documents, visited six CBS project sites, and interviewed 120 key stakeholders. Key themes that emerged included the need for greater clarity on the scope, purpose, and function of CBS and how data collected intersect with and is used within Indonesia’s broader early warning disease surveillance architecture, a need to harmonisation CBS legislation and implementation guidance across and within ministries, and that ongoing investment in essential health system building blocks – workforce, data systems, and analytical capacity – is required to ensure the scalability and sustainability of CBS across the country.

**Conclusions:**

Although CBS holds promise for strengthening public health security in Indonesia, key challenges related to common understanding, workforce capacity, data flow, and system governance must be addressed if the strategy is to make a meaningful contribution. Effective collaboration among government ministries, administrative levels, and community stakeholders is crucial for adapting CBS to balance local and national health security priorities, while ensuring stability, sustainability, and scalability across Indonesia’s diverse contexts.

Community-based surveillance (CBS) for emerging infectious diseases (EIDs) is a decentralised approach that engages local communities to swiftly detect and report public health events at their inception [1]. This strategy facilitates rapid responses, allowing the containment of outbreaks before they can escalate. Beyond its potential for early outbreak detection, CBS models may also foster local ownership of and responsibility for health security, leading to enhanced and scalable preparedness, prevention, and response efforts [1,2]. While various definitions of CBS exist, we adopted the one proposed by the World Health Organization (WHO), which describes it as ‘the systematic detection and reporting of events of public health significance within a community-by-community members’ [3].

A systematic literature review of CBS activities in ten Southeast Asian countries suggested that, at the time, there was no evidence that CBS was being systematically used in the region to provide early warnings of outbreaks [4]. The authors reported that the only systems identified were located in Malaysia and Indonesia, which were designed in response to a public health event and as an active case-finding exercise. They added that the discussion in the literature was qualitative and focussed on evaluating the structures and operations of CBS systems rather than their disease detection performance. Subsequent research by Mariner and colleagues [5] reported that, in the context of animal health, CBS has resulted in increased case detection in countries, including Indonesia, experiencing highly pathogenic avian influenza, and, as a result, a better understanding of the epidemiological situation has emerged. They added that where highly pathogenic avian influenza was absent and CBS was implemented, the method did not result in false positives and contributed to the overall epidemiological assessment that the country was disease-free.

Other researchers reported that CBS’s success is closely tied to community acceptance of CBS workers and how ‘nested’ these workers are within an existing emergency response system that is readily accepted [1,4,6,7]. Success is also attributed to maintaining high motivation among CBS workers, with motivation for participation commonly described in altruistic terms when related to human health-focussed systems [4,6,7] and in altruistic and economic security terms within the animal health domain [5,8]. Mariner and colleagues [5] also observed that clarity of surveillance objectives at the outset was essential to optimising CBS design to meet needs and that the quality of training programmes was associated with CBS workers’ level of competence in performing duties required with rigor. Tan and colleagues [7] added that a one-size-fits-all approach to community CBS training or in the recruitment of CBS is not appropriate, and CBS designers and implementers must consider different social and cultural contexts when addressing factors such as incentivisation, attitudes, and practicality.

Understanding the enablers and deterrents to CBS workers’ participation is not straightforward and differs across sectors and settings [4,8]. In the animal health domain, for example, studies have reported that concerns about economic consequences associated with disease outbreaks among livestock are a motivating factor for participation [8]. In contrast, in certain circumstances and settings, the fear of the consequences of reporting sick animals is a significant deterrent for farmers from raising an alert [9]. In the context of human health, the stigma or fear of social isolation associated with reporting an infectious disease within a household or small community can hinder the effectiveness of CBS [10].

Community ownership is often cited as a key factor in the reliable, stable, and comprehensive reporting of public health events [4,8,11–14]. Adopting context-appropriate communication and case reporting strategies has also been highlighted as important for building trust in reporting and facilitating timely data collection and exchange [15–19]. Crucially, a system’s effectiveness hinges on ensuring that the effort involved in collecting and reporting data brings perceived benefits to the CBS worker involved or to their immediate community.

The simplicity of data collection and reporting processes emerges as a key driver of CBS’s success, with technological solutions increasingly recognised for their potential to overcome reporting obstacles [7,18–20]. The WHO underscores this, emphasising the importance of technology in enhancing early detection of suspected cases in CBS systems [21].

Dedicated mentoring of CBS workers [13,14,22,23] and comprehensive training [6,12,13,16,22,24] have also been found to significantly influence workers’ motivation and the quality of data they collect. Efficient vertical and lateral integration of CBS within broader data systems has also been reported as crucial for long-term success and scalability. Notably, allocating human resources to facilitate lateral integration (*i.e.* between inter-ministry stakeholders operating at the same level) is recognised as an essential enabling factor [25].

In the wake of the COVID-19 pandemic, the Government of Indonesia reaffirmed its commitment to a whole-of-government/whole-of-community approach to bolster national preparedness for EID threats. Central to this agenda was the development of legislative instruments (*i.e.* laws, decrees, guidelines, and procedures) to drive a coordinated approach to early-warning human and animal disease outbreak surveillance, placing communities at the heart of the issue. Specific to this study, the Government of Indonesia’s Regulation No. 7 of 2022 [26] instructed relevant line ministries to use CBS to enhance the reach, sensitivity, and timeliness of outbreak-prone infectious disease surveillance.

To support the Government of Indonesia’s implementation of CBS, the Government of Australia, through the Australia-Indonesia Health Security Partnership commissioned research to guide CBS enhancement in the country. This research sought to provide evidence on which the Government of Indonesia may move forward with the prudent implementation of CBS in the country. Here, we report the findings of that research.

## METHODS

We used policy analysis, site visits, and interviews with key informants to gather the data.

### Policy analysis

We identified policy and programme documents concerning CBS implementation in Indonesia through discussions with key stakeholders and the trawling of relevant Government of Indonesia ministry websites. We applied a data extraction process with a predefined coding scheme, which included fields related to document metadata (date, issuing authority, version, class of policy document [decree, guideline, policy, procedure]), for whom the document was written, scope, and key policy or program directives included. Extracted data from each document was recorded in an Excel spreadsheet, Microsoft 365 Apps for Enterprises version (Microsoft, Redmond, Washington, USA). Last, we used content analysis to synthesise pertinent information, incorporating a review process with key informants to seek clarification if needed.

### Site visits and key informant interviews

We conducted site visits and webinar-based interviews to purposefully select animal health and human health CBS projects in three provinces in Indonesia with which the Australia-Indonesia Health Security Partnership had a relationship. Additionally, we conducted semi-structured key informant interviews with government and non-governmental agencies that have a stake in CBS in Indonesia. An unstructured interview guide facilitated group and individual discussions during site visits, webinars, and with key informants. The guide’s design allowed participants to direct the conversations while ensuring that key topics were covered. Participants received information about the research verbally and in writing, and we obtained their informed consent to collect, analyse, and report their contributions.

### Data analysis

**Figure 1:**
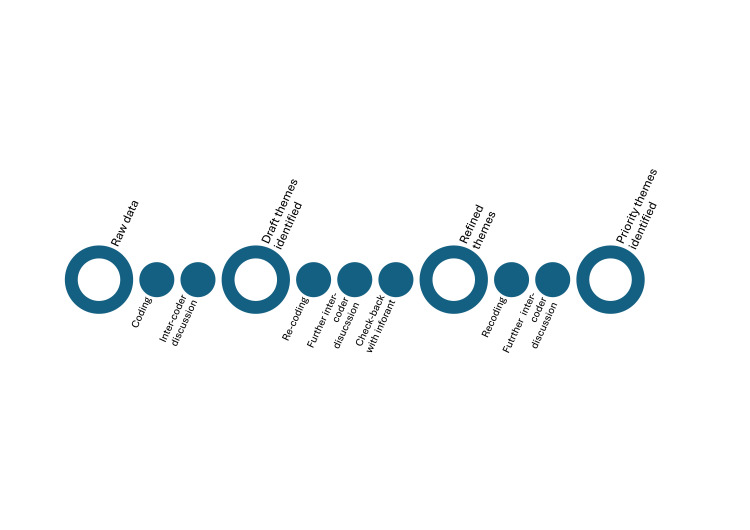
Illustration of the iterative approach used to analyse qualitative interview-based data.

We analysed the collected data using an inductive approach based on the work of Terry and colleagues [27]. This approach involved the iterative coding and categorisation of themes that emerged from the data using NVivo, version 14 (Lumivero, Denver, Colorado, USA). As data were collected from three heterogeneous sources, we allowed extensive time for iterative inter-coder discussion and interpretation of the raw data. We validated the findings from this process through a ‘check-back’ process with informants, whereby based on the data, themes, and their feedback, we determined the final thematic groups that reflected the dominant policy-relevant findings that emerged (**Figure 1**).

### Context and CBS system

Indonesia, a diverse archipelago nation comprising over 17 000 islands in Southeast Asia, is characterised by its rich cultural heritage, vast natural resources, and complex sociopolitical landscape. With more than 270 million inhabitants, it accounts for 3.2% of the world’s population. Politically, Indonesia is a presidential republic with a multiparty system. The president, elected by popular vote, is the head of the State and the Government.

The Indonesian health care system comprises a mix of public and private health care providers, with the government establishing policies and regulations. Outbreak-prone disease detection features in the work of the Ministry of Health (MoH) and the Ministry of Agriculture (MoA). The MoH’s early warning disease surveillance system – *Sistem Kewaspadaan Dini dan Respon* – routinely collects and analyses data from primary health centres (*puskesmas*), hospitals, and laboratories to detect events that have outbreak potential, while the MoA operates *Sistem Informasi Kesehatan Hewan Nasional Terpadu* that relies on farmers and community-based animal health workers to report sickness in animal stocks.

In response to lessons learned from the COVID-19 pandemic, the Government of Indonesia reaffirmed its commitment to detecting public health issues. The Government developed legislative measures that promote activity and coordination within and across human and animal health surveillance systems, with a focus on community engagement. Six critical legal instruments – including laws, presidential instructions, regulations, and guidelines – have been developed to enhance early warning disease surveillance.

## RESULTS

**Figure 2:**
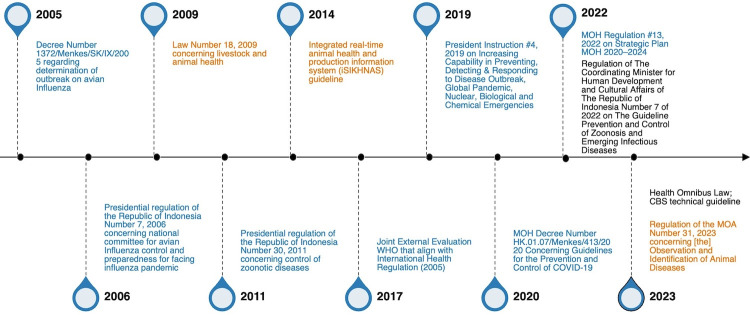
Key legal instruments related to community-based surveillance in Indonesia, 2005–23. Legal instruments in blue are related to human health, and those in orange are related to animal health. MOA – Ministry of Agriculture, MOH – Ministry of Health, WHO – World Health Organization.

During our policy analysis, we identified and reviewed eight legislative instruments related to CBS [26,28–34] (**Figure 2**).

We conducted interviews with 120 key stakeholders (**Table 1**). These included 99 district, subdistrict, and local animal and human health CBS stakeholders, and 11 staff members from international development assistance agencies with a stake in CBS in Indonesia. We conducted the interviews in Jakarta (the capital of Indonesia), during site visits to six CBS projects in two provinces, and during a webinar-based consultation with a seventh project in a third province (Bali) (**Table 2**).

**Table 1:** Key stakeholders interviewed by jurisdiction, Indonesia (n = 120)

	Sector				
	**Human health**	**Animal health**	**Wildlife health**	**Interagency coordination**	**Total**
**National**	5	2	2	1	10
**Provincial**	10	7		3	20
**District, subdistrict, and local**	55	22		2	79
**International organisations**					11
WHO					2
FAO					2
IFRC					3
USAID					3
US CDC, Indonesia					1

CDC – Centers for Disease Control and Prevention, FAO – Food and Agriculture Organization of the United Nations, IFRC – International Federation of the Red Cross, USAID – United States Agency for International Development, WHO – World Health Organization

**Table 2:** Summary of CBS projects

	System name	Health	Summary
**Central Java**			
Provincial level	Jogo Tonggo (Looking after your neighbors)	Human	Started during the COVID-19 pandemic. Encouraged residents to return to traditional community support values and work together to support one another during the pandemic, serving as a key risk communication and social support strategy. Provincial health office in Central Java proposes to use this model as the basis for future CBS implementation.
Demak	CBS for Leptospirosis vector control	Animal	Implemented by the district health office in Demak focussed on community awareness raising and vector control to prevent transmission of leptospirosis, a priority disease for the community. Does not include routine environmental or systematic cardinal sign/symptom monitoring and reporting mechanisms.
Boyolali	Red Cross CBS initiative	Human	Established in the Siswodipuran subdistrict before the COVID-19 pandemic. Implemented by the Indonesian Red Cross with support from various donors. Involves village volunteers actively seeking and reporting cases that display signs/symptoms of priority zoonotic diseases. Volunteers also report unusual events and provide broader community welfare support. Surveillance data are routinely reported to the Red Cross by a village coordinator using a phone application. If required, the coordinator informally shares information with the village leader and puskesmas verbally or through a WhatsApp group.
**South Sulawesi**			
Maros	Cadre-led CBS *Puskesmas Tompobulu*	Human	Villages cadres report suspected cases of noteworthy diseases to village midwives or the puskesmas *pembantu*/*pustu*, who assess the information and, if deemed necessary, report it to *puskesmas tompobulu*. None of the cadres have received training, and there is no evidence that case definitions are being used. While reporting processes appear to be well understood, protocols or standardised procedures to guide reporting were not evident.
	Red Cross CBS initiative	Human	Two villages, Tanete and Semangki, implement a more straightforward CBS system than that seen in the Siswodipuran Subdistrict, Boyolali, but with a broader range of diseases under surveillance. Volunteers report CBS data using a dedicated phone application. The same CBS model is being applied in 101 other villages in Maros by the district health office using health cadres. These cadres report the data they collect through the separate Ministry of Health developed phone application (i.e., a different phone application to the one used by those involved in the Red Cross CBS project implemented in Tanete and Semangki villages).
	*Pelapor Desa*	Animal	Community volunteers undertake active and/or passive surveillance for animal diseases near their villages. They report disease incidents to the district livestock office, which assesses and responds. Animal Health Centre (*puskeswan*) staff at the site visited report being outside of the *Pelapor Desa* reporting system.
**Bali**			
Buleleng	Tim Siaga Rabies	Animal	Established in response to the high incidence of rabies in Bali. Engages community members to report rabid dog sightings to a *puskeswan*, who may then request that the community member track and/or monitor the dogs’ health. After liaison between the local *puskeswan* and *puskesmas*, community members may also be engaged to support public risk awareness raising, promote dog vaccination, and encourage prompt action after a dog bite. Activities are supported using village budgets.

CBS – community-based surveillance

Our data analysis revealed seven dominant themes relevant to adopting CBS to enhance early warning surveillance for emerging infectious diseases in Indonesia.

### Guidance is needed to support operationalisation

Although state-issued legal instruments (*i.e.* policies and guidelines) provided a structure for action at the national to subdistrict level, we found that these instruments were not familiar to – and hence, not used by – staff at the grassroots level. Notably, *puskesmas* and *puskeswan* staff, along with village administrators, had limited awareness of the legislation and guidance for CBS, and hence tended to rely on advice from funders or technical partners.

I have no idea that CBS was coming from a central level and that there are requirements for us to do it; I thought it was a voluntary activity (…) – Puskesmas *leader*

This limited awareness of the central government’s CBS-related instructions at the local level emphasises the necessity for orientation and practical operational guidelines that help ensure consistent and harmonised CBS approaches, which in turn support the Government of Indonesia’s broader all-hazards health security objectives [32]. Interviewees felt that guidelines are required to outline training and support requirements for CBS workers, specify the compulsory syndromes to be surveyed, clarify the link between surveillance and response, and establish the administrative arrangements, such as funding arrangements, that underpin system stability.

### CBS as part of an all-hazards early warning approach

Most of the analysed CBS systems focussed on monitoring either a single or limited set of diseases, an approach attributed to the influence of vertically funded initiatives established before the implementation of the all-hazards-aligned national CBS guidelines in 2023 [32]. Although disease-specific approaches may yield favourable outcomes within their designated areas, they do not embrace or align with the all-hazards intent for CBS advocated for by the Government of Indonesia [32]. Highlighting this point, a central-level health official from the Government of Indonesia commented that there should not be multiple CBS models operating across the country, as this would lead to an overwhelming allocation of resources to support the initiative, making data management and use highly challenging.

CBS needs to feed into and support our core early warning surveillance system. *– Central-level Government of Indonesia health official.*

Raising this point is not intended to dismiss the relevance of disease-specific initiatives, but rather to highlight the emphasis participants placed on assessing the need for and structure of CBS within the broader context of national health security capacity-building in Indonesia.

Interviewees reported that CBS models differ between the human and animal health sectors due to their distinct operational contexts, diseases, reporting units, response mechanisms, and capabilities. They mentioned that animal health-focussed CBS tends to rely on farmers or village animal health workers to report illness in livestock. For these groups, interviewees noted that the motivation to report can be complex, with tensions between individuals’ sense of moral obligation sometimes clashing with concerns for the economic consequences and stigma that may result for themselves and their broader communities if their reporting leads to authorities culling or quarantining livestock, both of which impact livelihoods. This, we heard, differs from human-health CBS, where cases typically present at a health facility, allowing people to convey the signs and symptoms they are experiencing, and therefore, more nuanced risk assessments can be made.

### CBS must be framed as an extension of routine surveillance functions

A constant narrative that emerged during interviews suggested that, to be sustainable, CBS initiatives must be designed with an understanding of the budgetary, human resource, and logistical constraints faced at the periphery of human and animal health systems. Furthermore, many respondents considered that CBS should be incorporated into standard practices and add demonstrable value to justify the extra effort and work required to establish and operate it. Despite the ubiquity of this advice, we uncovered instances where CBS initiatives, motivated by an intent to ensure success, had introduced extra-national funded payments to CBS workers (*i.e.* stipends, often multiple times higher than that received by other community volunteers) to encourage sustained engagement, as well as parallel phone applications and new data flow processes. Both community and government interviewees raised questions about the ethics of this practice and its sustainability. Additionally, we encountered CBS systems primarily being used as a tool for behaviour change communication rather than early event detection, potentially diluting the core purpose of CBS and, as a government officer stated, ‘confusing people’.

Interviewees repeatedly commented on the disparities in resources available to the animal and human health sectors, which frames recognition of the inequitable operational landscapes in which each sector operates. All the people we interviewed agreed that a one-size-fits-all approach is not appropriate.

### Workforce and data systems are core to sustainable CBS systems

The CBS workers' pivotal role in ensuring the surveillance systems’ functionality, stability, and success is well documented in the literature. The data collected from interviews conducted for our study reaffirm this. Almost universally, interviewees highlighted the need for and importance of comprehensive training and support for CBS workers to ensure they could perform their duties. They emphasised the importance of structured training tailored to the specific responsibilities of CBS workers, along with ongoing supervision to ensure adherence to best practices. Clarifying tasks, methods, and duties was seen as crucial, as was emphasising the need for well-articulated implementation protocols.

We cannot be expected to understand what they (authorities) want from us; we need training. *– Community member.*

I am not a doctor, and I shouldn’t be responsible for making health decisions (i.e. differential diagnosis); but I’m happy, and I want to help my community; that’s why I did this. *– Community member.*

Furthermore, concerns were raised regarding the lack of external validation for many practices, which left some *puskesmas*, *puskeswan*, and CBS workers uncertain about the accuracy of their actions.

Our finding that CBS workers, who are typically non-professional volunteers, are sometimes tasked with complex decision-making responsibilities (*e.g.* screening symptoms and escalating reports) is noteworthy. There was a general sentiment among interviewees that skilled professionals should perform such tasks while CBS workers focus on process-driven data collection and reporting. In this context, they suggested that standardised protocols be developed and implemented to mitigate reliance on an individual CBS worker’s judgment. Similarly, there was concern that being overambitious with the scope of conditions under surveillance and the complexity of case definitions risks both stability and sustainability. Conversely, to ensure stability and sustainability, the designer of CBS systems must be mindful of the on-the-ground reality and tailor the systems accordingly.

We identified a preference for digital reporting systems, particularly through phone-based apps and platforms such as WhatsApp. These technologies were reported to facilitate citizen participation, streamline data transfer, and enhance communication. However, challenges were noted, such as parallel reporting and difficulty transferring data collected from one platform to another.

### Data analysis should be done where response decisions are made

Interview-based data collected during site visits revealed that although *puskesmas* (for human health) and subdistrict animal health offices (for animal health) typically designate an officer to manage surveillance data – including CBS-derived data – there was a feeling that these personnel often lack the expertise to generate epidemiologically useful intelligence or to integrate CBS data into routine surveillance practices. One human health officer noted that they collected CBS data. Still, the data did not align with the usual surveillance workflows or syndrome categories, so they were unsure how to handle the data. This comment suggested that data systems and analysis capabilities at the district/subdistrict levels must be enhanced if CBS data are to be used to generate meaningful information.

Interviewees suggested that building the capacity of local operators to use CBS data appeared necessary for several reasons. First, they indicated that the intricacies and dynamics of local contexts influenced the interpretation of surveillance data, emphasising the importance of analysts being familiar with the communities under surveillance. Second, they said that local analysis enhanced the timeliness of response by facilitating the rapid identification of emerging health threats. Third, they added that decentralising analysis aligned with the principles that underpin community-led surveillance. Fourth, they suggested that empowering local stakeholders by building capacity would foster ownership, which could support an increase in demand for quality data and data systems.

The interviewed senior health officials also called for broader use of CBS data, suggesting it should be leveraged to conduct temporospatial analyses of disease trends and patterns. This analytical approach, it was suggested, would aid the identification of new and evolving disease patterns across populations and over time, thereby enhancing the effectiveness of disease surveillance and response efforts.

### Existing governance and financing arrangements pose challenges

We found that details regarding the delineation of responsibilities for community-based activities between government ministries – accompanied by a range of non-harmonised operating mandates and directives – made CBS governance opaque, emphasising the need for local leadership and coordination. While Kemenko PMK had been delegated with this task, interviewees commented that the agency’s ‘lack of presence at the sub-national level’ and limited technical expertise and authority to direct action posed challenges to its effective performance in this role. Government interviewees, from local to national levels, reported the imperative of local government involvement to operationalise, integrate, and sustain CBS activities effectively, noting that the Ministry of Home Affairs was well-positioned to provide the necessary mandate to make this happen. Furthermore, interviewees suggested that the MoH and MoA (and Ministry of Environment and Forestry if surveillance were to include wildlife), which both have a mandate to implement and the required technical expertise in disease surveillance, should be responsible for developing technical standards and guidelines, providing training and mentoring, leading the operationalisation of CBS activities, and overseeing quality assurance.

Interviewees expressed divergent opinions about funding for village-level CBS activities, particularly given differing viewpoints on whether CBS workers should receive financial compensation for their service and, if so, from which budget. While a definitive solution remains elusive, they noted that CBS incurs costs that necessitate funds being allocated from certain quarters for its implementation, scaling, and maintenance.

### Rigorous performance monitoring is needed

While most interviewees considered CBS to hold promise, the absence of rigorous data hindered our assessment of the actual performance of existing systems. They acknowledged challenges in determining the sensitivity, specificity, and timeliness of CBS systems, given the absence of a ‘gold standard’ record of actual outbreaks against which their data could be compared. Model-based performance assessment approaches were suggested as a feasible alternative worth exploring in future work.

Interviewees reported that communities in Indonesia were used to volunteer-based social service delivery and valued it. For example, regarding CBS-based volunteerism, an interviewee involved in Jogo Tonggo in Central Java commented that their community members, who are participant volunteers, appreciated the initiative because it aligned with their values of caring for others and with the social structures, such as community women’s groups, which have been operating for many years. The interviewee commented that this linkage fostered the commitment and sustainability of the programme, as the volunteers placed personal value on their contributions and received ongoing positive feedback from their peers, which served to reinforce their practice.

The absence of a cost-benefit analysis comparing the costs of training, infrastructure, and ongoing support required for CBS against the benefits derived was also noted and deemed necessary, particularly by middle- and upper-level government officials. This sentiment was understandable given these groups’ responsibilities of managing budgets and making service delivery decisions.

## DISCUSSION

The proposal to introduce CBS as part of early warning surveillance architecture in Indonesia is a significant and potentially destabilising shift and, as such, should not be done without due consideration of the impact such a change may have on the stability and function of existing (and core) early warning mechanisms in place in Indonesia. To be functional and add surveillance value, CBS must fit within established and robust systems infrastructure that can support and sustain the community-based mechanisms and workforce on which the strategy relies. Further, systems must be in place to receive, verify and act on generated signals. This is not a small undertaking and, if to be sustained, requires careful planning, substantial investment and long-term support.

Scaling CBS to a national level will necessitate a multifaceted strategy that includes the alignment of complementary legal frameworks, policies, and protocols to guide implementation; ongoing investment in training, supervision, and data systems, including systems and infrastructure that support the integration of CBS data into broader EID intelligence generation; and mechanisms to ensure data and process quality are maintained. Furthermore, if CBS is to be a bottom-up initiative, it is crucial to monitor, capture, and report the added value of the surveillance strategy for end-users, including the community and the workers who directly support them. Achieving these fundamentals across Indonesia’s diverse settings will require significant effort, time, and resources. The importance of these investments cannot be overstated if the Government of Indonesia’s vision for CBS is to be realised, scaled, and sustained in the long term. Development partners are encouraged to focus their support on building the foundational elements of CBS's enterprise architecture.

Given the size and complexity of Indonesia’s human and animal health sectors, as well as the inherent risks of prematurely introducing new surveillance methods into often fragile systems, a staged approach to the CBS roll-out is recommended. For example, establishing CBS demonstration sites within each province can serve as a strategy to test, prove value, and refine the model locally before scaling. This approach will allow authorities to showcase CBS, thereby building familiarity and demand for its use at district and subdistrict levels.

While CBS is conceived as a surveillance strategy, our findings indicate that interviewees often framed it as fostering community engagement and promoting health. These dual roles – epidemiological monitoring and community mobilisation – are not inherently incompatible, but in practice can be challenging to reconcile. Without an articulated and shared understanding of CBS’s core function, there is a risk that its epidemiological purpose becomes diluted. This ambiguity can undermine the consistency and quality of surveillance efforts, making it more difficult for health authorities to manage, explain, and evaluate system performance across diverse contexts.

Objective and standardised metrics to measure the effectiveness of CBS were unavailable; therefore, the study relies on qualitative opinion-based data. Without well-defined indicators (e.g., sensitivity, specificity, stability, timeliness), it remains unclear if, and how, CBS adds value to early warning disease outbreak detection in Indonesia. Well-established and widely used tools, such as German and colleagues’ 2001 guidelines for evaluating public health surveillance systems [35], provide both a framework and practical guidance on what should be considered when monitoring a surveillance system's performance and how to measure it. These (or similar) guidelines should inform the MoH’s development of a comprehensive monitoring, evaluation, and learning framework to be implemented alongside CBS’s rollout.

The absence of robust performance metrics for CBS is a significant concern and likely limitation to its ongoing development. While we report interviewees’ recommendations to use model-based approaches to estimate performance, we suggest a comprehensive evaluation framework. This framework could include core indicators spanning sensitivity, timeliness, data completeness, and process indicators, including reporting consistency, community participation rates, and volunteer retention. To contextualise CBS performance, comparisons could be made with established surveillance systems, such as the *System Kewaspadaan Dini dan Respons*, animal health reports, or hospital-based surveillance, where data are available. Baseline capacities, such as training programs, supervision mechanisms, and digital reporting tools, should also be considered in interpreting CBS’s sustainability. 

Our findings highlighted the relatively large number of line ministries involved in the implementation, governance, and support of CBS in Indonesia, particularly at the local level. The suggestion that local governments, which have the mandate to direct and manage action at the community level, play a key role in the coordination of CBS activities while technical oversight is provided by the MoH and MoA (for human health and animal health CBS, respectively) seems reasonable, but will need to be tested within the national context. This suggestion aligns with existing literature [8]. Lessons from other initiatives, such as village health worker initiatives, offer valuable insights that CBS system designers can build upon. Regardless of the adopted governance arrangements, it is crucial to ensure that local CBS systems are implemented in a manner that supports the national goal of enhancing all-hazards health security in Indonesia. Articulating this goal and demonstrating how local CBS activity can address local needs and contribute to national health security strengthening ambitions aligns with existing literature [7,8], and is crucial if the CBS model is to be operationalised in the highly decentralised Indonesian health system.

Our research supports the conclusion of prior research [1,4,6,7] that the success of CBS data collection is tied to communities’ sense of value in the activity and acceptance of CBS workers, particularly if these workers are integrated within an existing emergency response system that is well-accepted. Our results also suggest that successful implementation can be attributed to the high motivation among CBS workers, who, based on our sample, describe their drive to participate as being related to the ‘giving of service’ to their communities. System fragility, we found, was associated with an overly ambitious number and complex case definition, as well as cumbersome reporting processes.

While the provision of material incentives (in the form of funds to cover time and out-of-pocket expenses) was noted as a motivation, so too was access to opportunities to increase knowledge through training, a sense of companionship among CBS workers, shared responsibility for outcomes, and feeling of personal responsibility of the programme’s success. CBS’s workers’ motivations are likely to differ between groups and across regions. They will also likely change over time. As such, understanding what motivates people to volunteer and collect data for a CBS system will require ongoing qualitative research, with the findings used to inform CBS worker recruitment and retention strategies.

Our finding that CBS models differ between the human and animal health sectors is essential, given the Government of Indonesia’s intention to align the sectors’ disease surveillance and create opportunities for enhanced detection of emerging and outbreak-prone zoonotic diseases. Understanding the similarities and differences between these two sectors will help conceptualise how the two sectors’ surveillance activities (and data) may be integrated for mutual benefit. Further, this approach aligns with Indonesia’s commitment to take a One Health approach, and many will watch Indonesia keenly as countries worldwide seek practical examples of operationalising a One Health approach for EID detection. The Government of Indonesia can support global learning by sharing its experience with CBS with its neighbours through groups, including the Association of Southeast Asian Nations and the WHO.

There is vehement agreement among stakeholders that technology is a lynchpin for CBS’s scalability. Given that implementing digital data collection, reporting, and analysis platforms enhances the efficiency and accuracy of surveillance processes, exploring technological solutions suitable to various geographical and infrastructural contexts will be required; this aligns with the sentiment expressed in the existing literature [7]. The Indonesian MoH’s commitment to a national ‘digital reform agenda’ [36] and its demonstrated familiarity with and use of communication technology in health (and, more broadly, society) indicate the country’s readiness to adopt electronic tools. The prudent adoption of the growing number of readily accessible digital applications – including data collection, visualisation, analytical, and communication tools – should be considered, as should the readiness of the health system and its users to adopt, integrate, and ultimately benefit from their use. These observations, while centred on the Indonesian experience, are likely universally relevant, and as such, the lessons learned in Indonesia may inform the digitisation of enhanced surveillance systems' functionality elsewhere.

The ethical implications of CBS implementation deserve careful consideration, particularly concerning financial incentives and the delegation of symptom recognition and reporting tasks to community members. These practices raise important issues, including equity, voluntarism, and the limits of task-shifting. While financial incentives may help motivate and reduce opportunity costs for community workers, they can also unintentionally undermine intrinsic motivation or create dependence on external funding, raising concerns about long-term sustainability. Similarly, relying on laypersons to perform quasi-clinical functions, such as identifying disease symptoms, can blur professional boundaries and lead to questions about training adequacy, responsibility, and data quality. Drawing on principles from community-based participatory research, CBS programs must ensure that CBS workers’ roles are clearly defined, expectations are transparent, and safeguards are in place to prevent the exploitation of community labour. Equitable participation requires that communities not only contribute to surveillance efforts but also be beneficiaries of the systems they help sustain. Incorporating ethical oversight mechanisms and involving communities in the design and governance of CBS can help balance operational efficiency and ethical integrity.

This research was conducted as part of the Australia-Indonesia Health Security Partnership, an initiative supported by Australian Aid. In addition to providing the review team and managing field logistics, the Partnership was actively engaged in various health-related projects at each study site. This dual role may have influenced participants' perceptions of the research team and the interview process. Specifically, the Partnership’s visible presence and operational support may have contributed to an implicit power imbalance, potentially affecting the degree to which interviewees felt comfortable speaking freely. In settings where deference to authority and norms of politeness are culturally reinforced, this may have introduced social desirability bias or prompted strategic responses. While difficult to quantify, such dynamics are common in field-based qualitative research, particularly where researchers are affiliated with donor-funded programs. In future studies, incorporating participatory or co-design elements may empower communities and reduce asymmetries. While such measures cannot eliminate the influence of donor affiliations or social expectations, they represent steps in fostering more open and authentic dialogue in qualitative research. 

The One Health framework offers an operational platform for fostering convergence across human, animal, and environmental health systems. In Indonesia, where zoonotic disease threats such as avian influenza, rabies, and leptospirosis persist, the potential for One Health approaches to enhance surveillance, outbreak response, and risk reduction is particularly relevant. Opportunities lie in leveraging existing multisectoral collaborations, such as the National Zoonoses Committee, and integrating data and expertise across ministries. The decentralised governance structure in Indonesia also provides entry points for locally adapted One Health interventions. However, significant barriers remain. Institutional silos, misaligned policy mandates, and limited intersectoral data sharing impede coordination. Capacity disparities between sectors, particularly in the veterinary and environmental health sectors, further constrain joint planning and implementation. Sustainable funding mechanisms and legal frameworks to formalise cross-sectoral collaboration are also underdeveloped [37,38]. Despite these challenges, Indonesia's commitment to adapting One Health approaches to intersectoral disease risk management is exemplified through the establishment of the national One Health Coordinating Unit and a National Zoonosis Committee, the country’s development of a One Health Joint Action Plan, and its championing of the Association of Southeast Asian Nations regional One Health Joint Plan of Action which seeks to foster collaboration across human, animal, plant, and environmental health among the member States [39–41]. The Government of Indonesia’s national and regional leadership for One Health raises hope for a more integrated administrative and operational approach to address health security threats at their source across the region.

### Limitations

This study has several limitations. First, the analysis was based on data collected from a limited number of CBS projects, potentially missing insights from other locations or projects. Second, the observed CBS projects were initiated before national guidelines for all-hazards CBS were disseminated. This might mean that these projects do not align with the vision for CBS that the Government of Indonesia aspires to. Third, the lack of available quantitative data on the CBS systems’ performance meant the review team had to rely on the stakeholders’ views, which could introduce bias. Fourth, the role of the Australia-Indonesia Health Security Partnership should be noted; not only did it commission and facilitate this study and house a review team member, but it also supports a range of projects within each study site that may have influenced how candid interviewees felt they could be. Finally, interviews with stakeholders from Bali were conducted via video conference, which may have resulted in some nuances in their statements being overlooked.

## CONCLUSIONS

CBS offers an undeniable opportunity to elevate public health security in Indonesia. To realise its potential, addressing challenges such as human resources, data quality, and governance is imperative. It is now essential to seed and encourage collaboration between ministries, different levels of government, and communities to tailor CBS models to be responsive to the needs and diverse contexts of animal and human health across Indonesia.

## References

[1] McGowan CR Takahashi E Romig L Bertram K Kadir A Cummings R Community-based surveillance of infectious diseases: a systematic review of drivers of success. BMJ Glob Health. 2022;7:e009934. 10.1136/bmjgh-2022-009934 35985697 PMC9396156

[2] Ratnayake R Tammaro M Tiffany A Kongelf A Polonsky JA McClelland A People-centred surveillance: a narrative review of community-based surveillance among crisis-affected populations. Lancet Planet Health. 2020;4:e483–95. 10.1016/S2542-5196(20)30221-7 33038321 PMC7542093

[3] Technical Contributors to the June 2018 WHO meeting A definition for community-based surveillance and a way forward: results of the WHO global technical meeting, France, 26 to 28 June 2018. Euro Surveill. 2019;24:1800681. 30646977 10.2807/1560-7917.ES.2019.24.2.1800681 PMC6337056

[4] Halton K Sarna M Barnett A Leonardo L Graves N A systematic review of community-based interventions for emerging zoonotic infectious diseases in Southeast Asia. JBI Database Syst Rev Implement Reports. 2013;11:1–235. 10.11124/01938924-201311020-00001 27820440

[5] Mariner JC Jones BA Hendrickx S El Masry I Jobre Y Jost CC Experiences in participatory surveillance and community-based reporting systems for H5N1 highly pathogenic avian influenza: a case study approach. EcoHealth. 2014;11:22–35. 10.1007/s10393-014-0916-0 24643858 PMC4046079

[6] Merali S Asiedu-Bekoe F Clara A Adjabeng M Baffoenyarko I Frimpong JA Community-based surveillance advances the global health security agenda in Ghana. PLoS One. 2020;15:e0237320. 10.1371/journal.pone.0237320 32780775 PMC7418973

[7] Tan YR Nguyen MD Mubaira CA Kajungu D Kumar D Canlas FC Building citizen science intelligence for outbreak preparedness and response: a mixed-method study in nine countries to assess knowledge, readiness and feasibility. BMJ Glob Health. 2024;9:e014490. 10.1136/bmjgh-2023-014490 38508584 PMC10952866

[8] Azhar M Lubis AS Siregar ES Alders RG Brum E McGrane J Participatory disease surveillance and response in Indonesia: strengthening veterinary services and empowering communities to prevent and control highly pathogenic avian influenza. Avian Dis. 2010;54:749–53. 10.1637/8713-031809-Reg.1 20521726

[9] MacPhillamy IBJ Nunn MJ Barnes TS Bush R Toribio J-ALML Striving for long term sustainability — Is it time we changed our approach to animal health in low- and middle-income countries? Acta Trop. 2023;244:106946. 10.1016/j.actatropica.2023.106946 37236333

[10] Byrne A Nichol B A community-centred approach to global health security: implementation experience of community-based surveillance (CBS) for epidemic preparedness. Glob Secur Health Sci Policy. 2020;5:71–84. 10.1080/23779497.2020.1819854

[11] Valdez D Keys H Ureña K Cabral D Camilo F Ogando EC Malaria outbreak response in urban Santo Domingo, Dominican Republic: lessons learned for community engagement. Rev Panam Salud Publica. 2020;44:e92. 10.26633/RPSP.2020.92 32774350 PMC7406124

[12] Clara A Do TT Dao ATP Tran PD Dang TQ Tran QD Event-Based Surveillance at Community and Healthcare Facilities, Vietnam, 2016–2017. Emerg Infect Dis. 2018;24:1649–58. 10.3201/eid2409.171851 30124198 PMC6106426

[13] Kisanga A Abiuda B Walyaula P Losey L Samson O Evaluation of the functionality and effectiveness of the CORE Group Polio Project’s community-based acute flaccid paralysis surveillance system in South Sudan. Am J Trop Med Hyg. 2019;101:91–9. 10.4269/ajtmh.19-0120 31760972 PMC6776096

[14] Stone E Miller L Jasperse J Privette G Diez Beltran JC Jambai A Community event-based surveillance for Ebola Virus Disease in Sierra Leone: implementation of a national-level system during a crisis. PLoS Curr. 2016;8:8. 28123860 10.1371/currents.outbreaks.d119c71125b5cce312b9700d744c56d8 PMC5222551

[15] Metuge A Omam LA Jarman E Njomo EO Humanitarian led community-based surveillance: case study in Ekondo-titi, Cameroon. Confl Health. 2021;15:17. 10.1186/s13031-021-00354-9 33771200 PMC7995751

[16] Clara A Ndiaye SM Joseph B Nzogu MA Coulibaly D Alroy KA Community-based surveillance in Côte d’Ivoire. Health Secur. 2020;18:S23–33. 10.1089/hs.2019.0062 32004127

[17] Ezenyeaku C Nnebue C Nwabueze S Ezenyeaku C Udedibia I Iloghalu I Completeness of reporting in the community-based disease surveillance and notification system in Anambra State, Nigeria. Am J Public Health Res. 2020;8:77–86.

[18] Craig AT Beek K Godinho MA Ansari S Jonnagaddala J Asgari-Jirhandeh N Digital health and universal health coverage: opportunities and policy considerations for Pacific Island health authorities. Pacific Health. 2023;6:64. 10.24135/pacifichealth.v6i.64

[19] Craig AT, Beek K, Godinho MA, Ansari S, Jonnagaddala J, Linhart C, et al. Digital Health and Universal Health Coverage: Opportunities and Policy Considerations for Pacific Island Health Authorities. New Delhi, India: World Health Organization Regional Office for South-East Asia; 2022. Available: https://apo.who.int/publications/i/item/digital-health-and-universal-health-coverage-opportunities-and-policy-considerations-for-pacific-island-health-authorities. Accessed: 20 December 2024.

[20] Fadillah A Suroso AI Indrawan D How mobile technology can be used to develop real-time animal disease surveillance in Indonesia? Atlantis Press. 2019;19:132–136. 10.2991/isessah-19.2019.1

[21] Hemingway-Foday JJ Ngoyi BF Tunda C Stolka KB Grimes KEL Lubula L Lessons learned from reinforcing epidemiologic surveillance during the 2017 Ebola outbreak in the Likati District, Democratic Republic of the Congo. Health Secur. 2020;18:S81–91. 10.1089/hs.2019.0065 32004132

[22] Cox J Dy Soley L Bunkea T Sovannaroth S Soy Ty K Ngak S Evaluation of community-based systems for the surveillance of day three-positive Plasmodium falciparum cases in Western Cambodia. Malar J. 2014;13:282. 10.1186/1475-2875-13-282 25052222 PMC4110522

[23] Amhara National Regional State Health Bureau. Ethiopia, Amhara Region Infectious Disease Surveillance (AmRids) Project Completion Report. Bahir Dar, Ethiopia: Amhara National Regional State Health Bureau; 2015. Available: https://openjicareport.jica.go.jp/pdf/12234761_01.pdf. Accessed: 4 April 2025.

[24] Larsen TM Mburu CB Kongelf A Tingberg T Sannoh F Madar AA Red Cross volunteers’ experience with a mobile community event-based surveillance (CEBS) system in Sierra Leone during-and after the Ebola outbreak—a qualitative study. Health Prim Care. 2017;1:1–7.

[25] Van Boetzelaer E Chowdhury S Etsay B Faruque A Lenglet A Kuehne A Evaluation of community based surveillance in the Rohingya refugee camps in Cox’s Bazar, Bangladesh, 2019. PLoS One. 2020;15:e0244214. 10.1371/journal.pone.0244214 33362236 PMC7757896

[26] Coordinating Ministry for Human Development and Cultural Affairs. Coordinating Ministry of Human Development and Cultural Affairs. Regulation of The Coordinating Minister for Human Development and Cultural Affairs of The Republic of Indonesia Number 7 of 2022 on The Guideline Prevention and Control of Zoonosis and Emerging Infectious Diseases. Jakarta, Indonesia: Coordinating Ministry for Human Development and Cultural Affairs; 2022. Available: https://cdn.who.int/media/docs/default-source/searo/indonesia/permenko-no-7-tahun-2022-small-file.pdf. Accessed: 20 December 2024.

[27] Terry T, Hayfield N, Clarke V, Braun V. The SAGE Handbook of Qualitative Research in Psychology. In: Willig C, Rogers SW, editors. Thematic Analysis. California, USA: SAGE Publications Ltd; 2017. p. 17–36.

[28] Republic of Indonesia, Ministry of Health. [Law Number 17 of 2023 Concerning Health]. 8 August 2023. Available: https://peraturan.bpk.go.id/details/258028/uu-no-17-tahun-2023. Accessed: 20 December 2024. Indonesian.

[29] Republic of Indonesia. [Presidential Instruction Number 4 of 2019 Concerning Increasing Capabilities in Preventing, Detecting, And Responding to Disease Outbreaks, Global Pandemics, and Nuclear, Biological, and Chemical Emergencies]. 17 June 2019. Available: https://peraturan.bpk.go.id/Details/110251/inpres-no-4-tahun-2019. Accessed: 20 December 2024. Indonesian.

[30] Republic of Indonesia, Ministry of Agriculture, Directorate General of Livestock and Animal Health Services. [Law Number 18 of 2009 concerning Animal Husbandry and Animal Health]. 4 June 2009. Available: https://peraturan.bpk.go.id/Details/38634/uu-no-18-tahun-2009. Accessed: 20 December 2024. Indonesian.

[31] Republic of Indonesia, Ministry of Health, Directorate General of Disease Prevention and Control. [Guideline for the Implementation of Post-Pandemic COVID-19 Collaborative Surveillance]. Jakarta, Indonesia: Republic of Indonesia, Ministry of Health, Directorate General of Disease Prevention and Control; 2023. Indonesian.

[32] Republic of Indonesia, Ministry of Health. Directorate General of Disease Prevention and Control. [Community-based surveillance technical guideline]. Jakarta, Indonesia: Republic of Indonesia, Ministry of Health. Directorate General of Disease Prevention and Control; 2023. Indonesian.

[33] Republic of Indonesia, Ministry of Agriculture. [Regulation Number 31, 2023 Concerning Observation and Identification of Animal Diseases]. 2023. Available: https://peraturan.go.id/files/permentan-no-31-tahun-2023.pdf. Accessed: 20 December 2024. Indonesian.

[34] Ministry of Agriculture of the Republic of Indonesia. [Regulation of the Republic of Indonesia No. 47 of 2014 Concerning the Control and Eradication of Animal Diseases]. Jakarta, Indonesia: Ministry of Agriculture of the Republic of Indonesia; 2014. Available: https://ditjenpkh.pertanian.go.id/uploads/download/peraturan-pemerintah-republik-indonesia-no-47-tahun-2014-tentang-pengendalian-dan-penanggulangan-penyakit-hewan-1658819596.pdf. Accessed: 13 July 2025. Indonesian.

[35] German RR Lee LM Horan JM Milstein RL Pertowski CA Waller MN Updated guidelines for evaluating public health surveillance systems: recommendations from the Guidelines Working Group. MMWR Recomm Rep. 2001;50 RR-13:1-35, quiz CE1-7. 18634202

[36] Republic of Indonesia, Ministry of Health. [Minister of Health Regulation Number 13 of 2022 Concerning Amendments to Minister of Health Regulation Number 21 of 2020 Concerning the Strategic Plan of the Ministry of Health for 2020-2024]. 16 June 2022. Available: https://peraturan.bpk.go.id/Details/212810/uu-no-13-tahun-2022. Accessed: 20 December 2024. Indonesian.

[37] Saminarsih DS, Tyas ASA, Espressivo A, Magdalena C, Kautsar F, Hafizon I, et al. White Paper: Indonesia's Health Sector Development (2024-2034): Designing a Future for Policy and Delivery. Jakarta, Indonesia: Center for Indonesia’s Strategic Development Initiatives; 2023. Available: https://cdn.cisdi.org/whitepaper/Main%20Book:%20White%20paper%20on%20Indonesia%E2%80%99s%20Health%20Sector%20Development%20(2024-2034).pdf. Accessed: 20 December 2023.

[38] Minister of Village, Development and Disadvantaged Regions. [Regulation No. 2 of 2024 Concerning Operational Guidelines for the Focus of Village Funds]. Jakarta, Indonesia: Minister of Village, Development and Disadvantaged Regions; 2024. Available: https://jdih.kemendesa.go.id/public/documents//82046516-54d7-4f93-9e16-4958a3d794dcPeraturan_Menteri_Desa_dan_Pembangunan_Daerah_Tertinggal_Nomor_2_Tahun_2024_tentang_Petunjuk_Operasional_atas_Fokus_Penggunaan_Dana_Desa_Tahun_2025.pdf. Accessed: 20 December 2024. Indonesian.

[39] World Health Organization. Building resilience: The Indonesia One Health joint plan of action. 25 January 2024. Available: https://www.who.int/indonesia/news/detail/25-01-2024-building-resilience--the-indonesia-one-health-joint-plan-of-action. Accessed: 20 December 2024.

[40] Association of South East Asian Nations. ASEAN leaders' declaration on One Health Initiatives. 2023. Available: https://asean.org/wp-content/uploads/2023/05/11-ASEAN-One-Health-Initiative-Declaration_adopted.pdf. Accessed: 20 December 2023.

[41] Association of South East Asian Nations. ASEAN One Health Joint Plan of Action (2023-2030). 2 September 2023. Available: https://isomer-user-content.by.gov.sg/18/9bcf2c5f-79a3-4108-b350-8adfce2e54a9/ASEAN%20OH%20JPA_AHMM%20endorsed.pdf. Accessed: 20 December 2023.

